# Algae-Based Nanoparticles for Oral Drug Delivery Systems

**DOI:** 10.3390/md22030098

**Published:** 2024-02-21

**Authors:** Eliyahu Drori, Dhaval Patel, Sarah Coopersmith, Valeria Rahamim, Chen Drori, Suchita Suryakant Jadhav, Roni Avital, Yaakov Anker, Aharon Azagury

**Affiliations:** Department of Chemical Engineering, Ariel University, Kiryat Hamada 3, Ariel 4070000, Israelkobia@ariel.ac.il (Y.A.)

**Keywords:** algae, nanoparticles, bioadhesion, biomimicry, oral drug delivery, cellular uptake, mucoadhesion

## Abstract

Drug administration by oral delivery is the preferred route, regardless of some remaining challenges, such as short resident time and toxicity issues. One strategy to overcome these barriers is utilizing mucoadhesive vectors that can increase intestinal resident time and systemic uptake. In this study, biomimetic nanoparticles (NPs) were produced from 14 types of edible algae and evaluated for usage as oral DDSs by measuring their size, surface charge, morphology, encapsulation efficiency, mucoadhesion force, and cellular uptake into Caco-2 cells. The NPs composed of algal materials (aNPs) exhibited a spherical morphology with a size range of 126–606 nm and a surface charge of −9 to −38 mV. The mucoadhesive forces tested ex vivo against mice, pigs, and sheep intestines revealed significant variation between algae and animal models. Notably, *Arthospira platensis* (i.e., Spirulina) NPs (126 ± 2 nm, −38 ± 3 mV) consistently exhibited the highest mucoadhesive forces (up to 3127 ± 272 µN/mm²). Moreover, a correlation was found between high mucoadhesive force and high cellular uptake into Caco-2 cells, further supporting the potential of aNPs by indicating their ability to facilitate drug absorption into the human intestinal epithelium. The results presented herein serve as a proof of concept for the possibility of aNPs as oral drug delivery vehicles.

## 1. Introduction

Oral drug administration is the intake route preferred by patients and healthcare providers [1]. Oral delivery is non-invasive, painless, easy to perform, and can be self-administered [2]. Over the years, various NPs have been used as oral DDSs, such as polymeric NPs [3,4], liposomes [5], membrane-based NPs [6], nano-sized hydrogels [7], and recently biomimetic NPs derived from edible plants (e.g., corn [8], grapefruit [9], and ginger [10]). Yet, oral drug delivery faces many challenges that affect its efficiency, such as poor penetration, low solubility, enzymatic degradation, and short resident time (due to intestinal peristaltic movement), resulting in low bioavailability [11,12].

To overcome the low residence time of DDSs in the gastrointestinal tract (GIT), researchers have utilized floating DDSs (mainly suitable for delivery in the stomach [12]) and mucoadhesive DDSs. Mucoadhesive DDSs adhere to the intestinal mucous, prolonging their resident time and thereby improving systemic uptake [13]. For example, Rosso et al. employed bioadhesive chitosan sponges to extend intestinal residence time to 6 h from the 3 h observed without chitosan [14]. Moreover, Reineke et al. found that coating non-adhesive NPs with a mucoadhesive polymer increased their systemic uptake post-oral administration in mice from 6% to 67% [15]. Cheng et al. utilized chitosan-coated NPs for insulin delivery, revealing a 16-fold increase in bioavailability compared to free insulin administration [16]. Furthermore, mucoadhesive DDSs offer an option for localized treatment of intestinal disorders such as inflammation (e.g., Crohn’s disease and colitis) [17] and diverse forms of cancer [18]. Nevertheless, high mucoadhesion does not guarantee high systemic uptake.

Several mucoadhesive natural biopolymers (e.g., alginate [19], carrageenan [19], and fucoidan [20]) are major components in algae. Thus, we used the biomimetic approach and produced NPs directly from algae, assuming it would impart these NPs with mucoadhesiveness [21]. Additionally, edible algae are of great interest to drug delivery research due to their inherent biodegradability, non-immunogenicity, and biodegradability [22]. The main working hypothesis was that algal-based NPs would possess the mucoadhesion properties of the “parent” alga, making them great candidates to serve toward oral DDSs. Thus, our primary goal was to investigate the feasibility and effectiveness of biomimetic NPs derived from edible algae toward novel oral DDSs. To this end, the first objective was to extract NPs from the components of 14 different types of edible algae and characterize these algal NPs (aNPs) via cryo-TEM, dynamic light scattering (DLS), a texture analyzer, and FTIR analysis. The next objective was to identify aNPs with optimal characteristics for oral DDSs based on the aNP encapsulation efficacy and release profile, focusing on those with the highest mucoadhesion. The last objective was to evaluate whether aNPs with high mucoadhesion can efficiently penetrate Caco-2 cells—the standard in vitro model of the human intestinal barrier [23].

## 2. Results and Discussion

### 2.1. Characterization of aNPs

First, 14 different types of algae were used to produce 14 aNPs. These aNPs were analyzed for size, concentration, and protein content (see Table 1).

As can be seen in Table 1, the aNP size ranged from 126 to 605 nm, and the singular peak was detected (Figure S1). The relative aNP concentration varied between 1.5 to 60.8 × 10^9^ NPs/(mL × g), while the protein concentration ranged from 0.01 to 3.24 mg/mL. aNPs from *C. chamissoi* showed the largest particle size, 605 ± 67 nm, compared to *A. platensis*, which had the smallest particle size, 126 ± 02 nm. The concentration comparison showed a conflicting result: for *C. chamissoi*, the lowest relative concentration was obtained at 1.5 ± 0.3 × 10^9^ NPs/(mL × g), and for *A. platensis*, the highest relative concentration was significantly obtained at 60.8 ± 2.9 × 10^9^ NPs/(mL × g). Additionally, there were differences in protein content between the different algae: the aNPs with the highest protein content were those derived from *H. pluvialis* (i.e., 3.24 ± 0.02 mg/mL), followed by *A. platensis* with 2.61 ± 0.81 mg/mL. In all other aNPs, the protein concentration was lower than 1 mg/mL.

As mentioned, *A. platensis* NPs had the smallest particle size of 126 ± 2 nm among the tested algae (Table 1). Nano-sized DDSs are advantageous for cellular uptake via endocytosis. As shown in Table 1, the PDI values obtained for aNPs varied from 0.19 to 0.48. As the main objective was to identify the aNPs with the highest mucoadhesion, all aNPs were prepared under the same protocol. Further experiments should be employed to optimize the chosen aNP size and polydispersity (i.e., PDI value) via methods such as the LiposoFast device or optimizing ultrasonic/homogenizer parameters. Also, *A. platensis* NPs displayed the highest initial concentration of 60.8 ± 2.9 × 10^9^ NPs/(mL × g) (Table 1), indicating a substantial yield and cost-effective production process.

**Figure 1:**
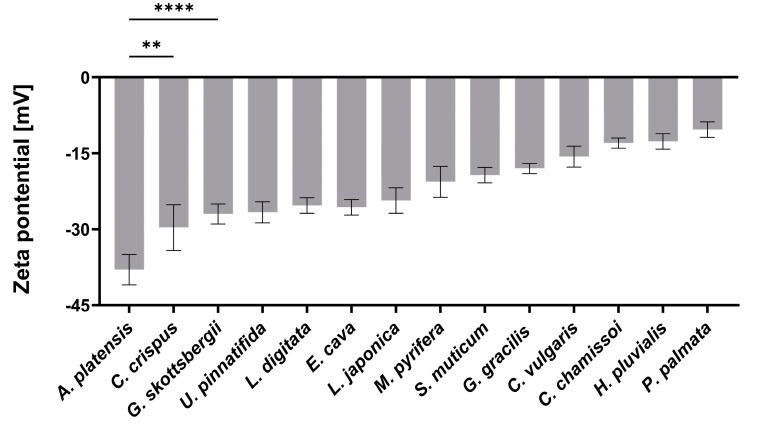
The measured zeta potential in DDW of the DDSs composed of the aNPs. An ANOVA test was conducted to ascertain the statistical significance of the measured zeta potential compared to *A. platensis* NPs. The presented data represent the mean ± SD *n* = 3, with statistical significance denoted as ** *p* < 0.01 and **** *p* < 0.0001 of aNPs compared to *A. platensis* NPs.

The surface charge (i.e., zeta potential) of NPs is another crucial property for predicting the effectiveness of an oral DDS [24]. Thus, the zeta potentials of the aNPs were measured in DDW for the DDSs composed from aNPs. The results are presented in Figure 1 below.

As depicted in Figure 1, the zeta potential values observed for the aNPs range from −38 to −9 mV, reflecting variety in the composition of components from the tested aNPs. Among these, *A. platensis* NPs displayed the most negative zeta potential of −38 ± 3 mV, while *P. palmata* NPs exhibited a nearly neutral surface charge of −9 ± 3 mV. Previously, it was shown that negative surface charge correlates with mucoadhesiveness by forming hydrogen bonds with mucins [25]. Another study showed mucins can attach to negatively charged molecules via positively charged amino acids in the terminal domains [26]. Yet, it was also demonstrated that loose mucins in the lumen adhere to and coat charged NPs and may neutralize their effective surface charge [27].

**Figure 2:**
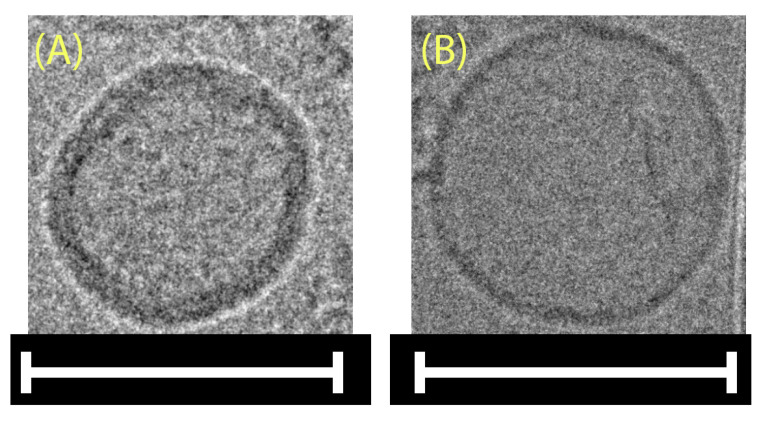
Cryo-TEM images of *A. platensis* NPs (**A**) immediately after production and (**B**) post-lyophilization, resuspension, and mild sonication. Scale bars: 100 nm.

Utilizing cryo-TEM (Figure 2) to confirm the spherical shape of *A. platensis* NPs was necessary since DLS size analysis relies on the assumption that the measured NPs are spherical. Additionally, the aNPs were lyophilized, resuspended in water, and imaged in cryo-TEM to further understand the formation mechanism (self-assembly).

It can be seen in Figure 2A,B that a spherical morphology was obtained in both cases. The reformation of spherical-shaped NPs post lyophilization and resuspension, as seen in Figure 2B, indicates that their formation is favored thermodynamically, as in the self-assembly mechanism. To further support this claim, FTIR analysis was applied in order to detect membrane lipids within the aNPs (Figure S2). The resulting spectra featured characteristic absorption bands indicative of membrane lipids, including the C-H stretching vibrations observed in the 2800–3000 cm^−1^ region and the C=O stretching of ester groups near 1800 cm^−1^ [28,29]. These findings corroborate the presence of lipid constituents within the aNP structure, which are well known for their ability to self-assemble into a bilayer spherical shape—a liposomal structure (a hint of this bilayer could also be seen in Figure 2).

### 2.2. Mucoadhesive Force of the aNPs

**Figure 3:**
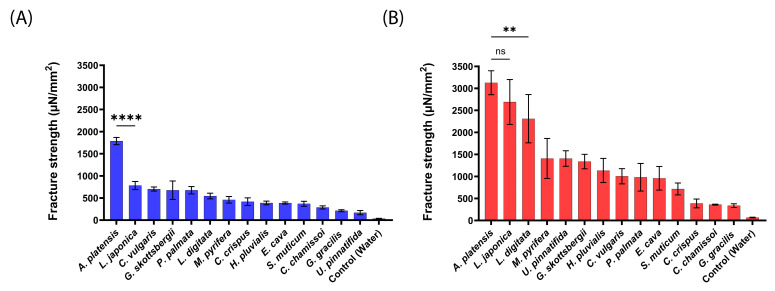
Mucoadhesion fracture strength of the produced aNPs against the small intestines of mice for (**A**) an applied force of 20 mN and (**B**) 200 mN. The ANOVA test was conducted to ascertain the statistical significance of the measured mucoadhesion fracture strength compared to *A. platensis* NPs. The values represent the mean ± SD of *n* = 4, where statistical significances are denoted as ** *p* < 0.01, **** *p* < 0.0001, and ns—nonsignificant.

**Figure 4:**
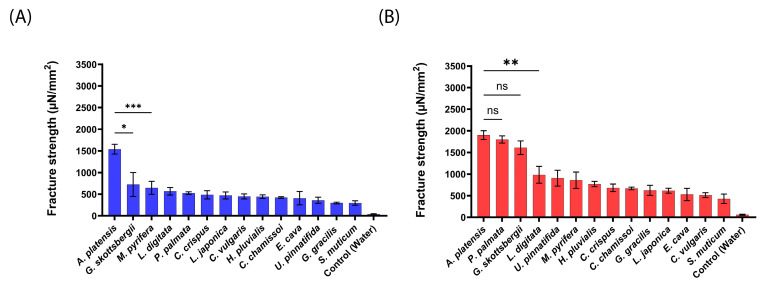
Mucoadhesion fracture strength of the produced aNPs against the small intestines of pigs for (**A**) an applied force of 20 mN and (**B**) 200 mN. The ANOVA test was conducted to ascertain the statistical significance of the measured mucoadhesion fracture strength compared to *A. platensis* NPs. The values represent the mean ± SD of *n* = 4, where statistical significances are denoted as * *p* < 0.05, ** *p* < 0.01, *** *p* < 0.001, and ns—nonsignificant.

**Figure 5:**
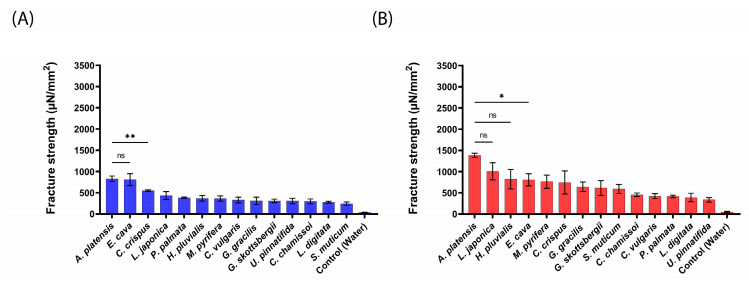
Mucoadhesion fracture strength of the produced aNPs against the small intestines of sheep for (**A**) an applied force of 20 mN and (**B**) 200 mN. The ANOVA test was conducted to ascertain the statistical significance of the measured mucoadhesion fracture strength compared to *A. platensis* NPs. The data represent the mean ± SD of *n* = 4, where statistical significances are denoted as * *p* < 0.05, ** *p* < 0.01, and ns—nonsignificant.

The mucoadhesion fracture strengths of the produced aNPs were measured against the small intestines of mice, pigs, and sheep using a texture analyzer. The obtained results are presented in Figure 3 (mice), Figure 4 (pigs), and Figure 5 (sheep).

As predicted and shown in Figure 3, *A. platensis,* NPs exhibited the highest mucoadhesion fracture strength of 1786 ± 81 and 3127 ± 272 µN/mm^2^ for 20 mN and 200 mN, respectively. These forces were 2–10-fold stronger than the other tested aNPs. *L. japonica* NPs also exhibited high mucoadhesion of 2691 ± 509 µN/mm^2^ at the applied force of 200 mN (statistically similar to *A. platensis* NPs). On the other hand, *U. pinnatifida* (170 ± 47 µN/mm^2^) and *G. gracilis* (338 ± 42 µN/mm^2^) aNPs exhibited the lowest mucoadhesion fracture strength towards the intestines of mice for both applied forces.

Generally, the measured mucoadhesive fracture strength increased when the applied force rose from 20 mN to 200 mN (Figure 3B vs. Figure 3A). We hypothesize that the increased applied force may augment the contact surface area by the disentanglement of mucins, thus increasing the number of surface interactions between the aNPs and the intestinal epithelial layer [30]. Also, since there is no clear standard regarding which animal model should be used for mucoadhesion measurements, the intestines of pigs were examined, as can be seen in Figure 4.

As shown in Figure 4 for pig intestines, *A. platensis* NPs exhibited the highest mucoadhesion fracture strength of 1539 ± 112 and 1901 ± 100 µN/mm^2^ for 20 mN and 200 mN, respectively. These forces were 2–5-fold stronger than observed for the tested aNPs. *P. palmata* and *G. skottsbergii* (1800 ± 83 and 1609 ± 158 µN/mm^2^, respectively) NPs also exhibited high mucoadhesion at the applied force of 200 mN (statistically similar to *A. platensis* NPs). On the other hand, *S. muticum* NPs showed the lowest mucoadhesion towards the intestines of pigs for both applied forces: 295 ± 52 and 427 ± 109 µN/mm^2^ for 20 mN and 200 mN, respectively.

As observed for mice intestines, a similar trend was detected herein for pig intestines. Enhancing the applied force from 20 mN to 200 mN (Figure 4B vs. Figure 4A) increased the measured mucoadhesive force. This observation further supports the hypothesis of an increase in contact area when the applied force increases. Next, the intestines of sheep were used to measure the mucoadhesion fracture strength of the composed from aNPs, as seen in Figure 5 below.

As shown in Figure 5 for sheep intestines, *A. platensis* NPs exhibited the highest mucoadhesion fracture strength towards the intestines of sheep, with 828 ± 66 and 1386 ± 46 µN/mm^2^ for 20 mN and 200 mN, respectively. These forces were 1.5–3.5-fold stronger than the rest of the tested aNPs. Notably, *L. japonica* and *H. pluvialis* (1008 ± 201 and 822 ± 230 µN/mm^2^, respectively) aNPs also exhibited high mucoadhesion at the applied force of 200 mN (statistically similar to *A. platensis* NPs). Conversely, *S. muticum* (243 ± 37 µN/mm^2^) and *U. pinnatifida* (335 ± 52 µN/mm^2^) aNPs exhibited the lowest mucoadhesion fracture strengths towards the intestines of sheep for both applied forces. Finally—as observed with mice and pig intestines—for all the tested aNPs, the measured mucoadhesion fracture strength was also increased when the applied force was increased from 20 mN to 200 mN.

**Figure 6:**
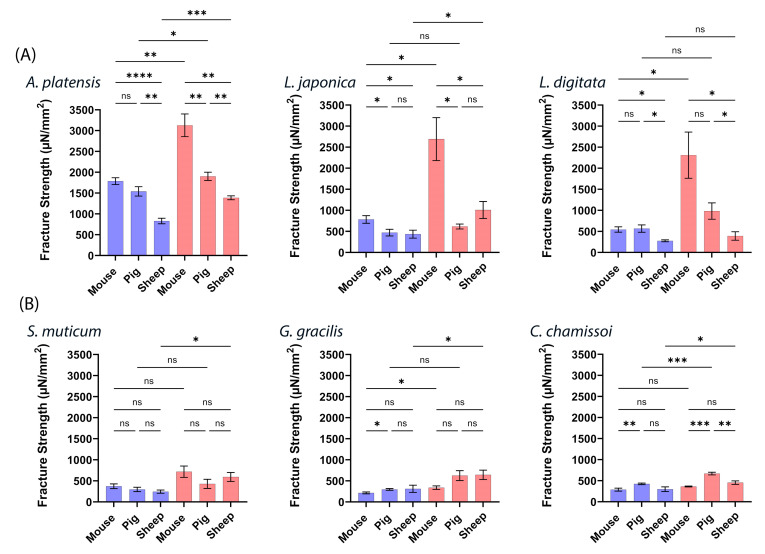
Mucoadhesive fracture strengths of six different aNPs with applied forces (20 mN—blue bars and 200 mN—red bars) in (**A**) three aNPs with the highest mucoadhesion and (**B**) three with the lowest. ANOVA test was conducted to ascertain the statistical significance of the measured mucoadhesion fracture strength. The data represent the mean ± SD of *n* = 4, where statistical significances are denoted as ns—nonsignificant, * *p* < 0.05, ** *p* < 0.01, *** *p* < 0.001, and **** *p* < 0.0001 for multiple-variable comparisons.

To further analyze and compare the three ex vivo intestinal animal models, we chose three aNPs with the highest mucoadhesion—*A. platensis*, *L. japonica*, and *L. digitata* —and three with the lowest—*S. muticum*, *G. gracilis*, and *C. chamissoi*. Note that the obtained mucoadhesion fracture strengths are categorized and color-coded by the applied forces of 20 (blue bars) and 200 mN (red bars) and presented in Figure 6 below.

Figure 6 illustrates notable variations in mucoadhesion observed among the *ex vivo* intestinal models for the highest and lowest aNPs. Figure 6A highlights the algae with the most robust mucoadhesive properties, with the mouse intestinal model yielding the highest results at 200 mN, followed by pig and sheep intestines. At 20 mN, no significant difference was observed between mucoadhesion in mice and pigs, but both outperformed sheep intestines. In contrast, the findings in Figure 6B are not as definite regarding aNPs with weaker mucoadhesive properties. *A. platensis* NPs consistently exhibited superior mucoadhesion, particularly at 200 mN (often at 20 mN).

### 2.3. Protein BLAST of Mucin2

Comparing the sequence of human Mucin2 (a protein consisting of 5130 amino acids (aa)) to that of the tested ex vivo animal models —mice, pig, and sheep intestines—provides valuable insights into the correlation between animal models and humans. Thus, to evaluate which intestinal animal model is the most suited to predict mucoadhesion in humans, we compared the sequences of the glycoprotein Mucin2, which is the main component of mucus [31]. The comparison was carried out via the Protein BLAST tool, and the results are presented in Table 2.

First, it is important to define the specific sequence alignment metrics of the Identities and Positives parameters mentioned in Table 2. Identities refer to the count of positions where the amino acids in compared sequences match precisely. Positives denote positions where the aligned amino acids exhibit similar properties (i.e., both are hydrophobic/acidic, etc.). As shown in Table 2, the mouse Mucin2 sequence displayed more Identities and Positives than the sheep and pig sequences, indicating the high resemblance of Mucin2 from a mouse to that from a human.

Moreover, Insertions cause “gaps” in the alignment, resulting in missing or incomplete information known as insertion–deletion mutations (indels). The mouse sequence of Mucin2 had no gaps, whereas the sheep and pig sequences displayed 3% gaps, indicating slight structural divergence in those regions. Additionally, query cover refers to the proportion of the query sequence (human Mucin2) that aligns with the compared sequence. Higher coverage implies a more significant overlap, potentially highlighting functional regions. Here, the sheep sequence exhibited the highest coverage (87%), the pig sequence had 60%, and the mouse sequence had the lowest coverage (43%).

The final score presented in Table 2 considers all the parameters and quantifies the extent of similarity between the compared sequences. The mouse sequence exhibited the highest score (2532), showing the highest likeness to human Mucin2. The sheep and pig sequences had similar final scores (1495 and 1474, respectively), signifying relatively lower but noteworthy similarity to human Mucin2.

Mucoadhesion results from interaction with mucus composed of mucins. Thus, comparing mucins could serve as an indication of mucoadhesion in humans. Since mice showed the highest similarity to human Mucin2, it could be suggested as the most suited animal model for studying mucoadhesive properties for oral drug delivery. Further supporting this conclusion is that mice were preferred for measuring mass transport across the mucosal layer of the intestines since their intestinal resident time and mucus thickness align well with human conditions [27].

However, many other parameters, such as intestinal pH and morphology (i.e., surface area), may affect mucoadhesion. Thus, we continued to compare mice intestines to human intestines. For example, the pH range in the human intestine is 6.0–7.4 [32], and for mice it is 6.3–8.1 [33]. Additionally, Stanford et al. have demonstrated marked similarities in developmental trends over time between murine and human systems [34]. A detailed comparison of the two intestines has revealed more similarities than differences [35]. Though these observations are insufficient to prove complete similarity, they contribute to the conclusion that mice intestines are an appropriate model for assessing mucoadhesion and oral delivery efficiency in humans.

### 2.4. Cellular Uptake of aNPs

To examine the uptake of aNPs into Caco-2 cells, fluorescein isothiocyanate dextran (FD40), a hydrophilic fluorescent molecule, was encapsulated in six different algae aNPs. FD40 was chosen for its traceability and similarity in size to therapeutic proteins such as erythropoietin (30.4 kDa) and peginterferon-α-2a (~40 kDa). Additionally, the fluorescent property of FD40 enabled the quantification of encapsulation efficiency (*EE*), as shown in Table 3.

Table 3 shows the *EE* percentages of six different types of aNPs with FD40. As can be seen, the observed *EE* values ranged from 32% to 55%. The calculation of the *EE* was performed using the following equation:

(1) $$EE=\left(1-\frac{m_{Supernatant}}{m_{0}}\right)\times 100\%$$

Since the aNPs possess a liposome-like structure, hydrophilic molecules (e.g., FD40) are expected to be encapsulated within their hydrophilic core. In contrast, lipophilic molecules would absorb into the hydrophobic envelope of the liposome [36].

**Figure 7:**
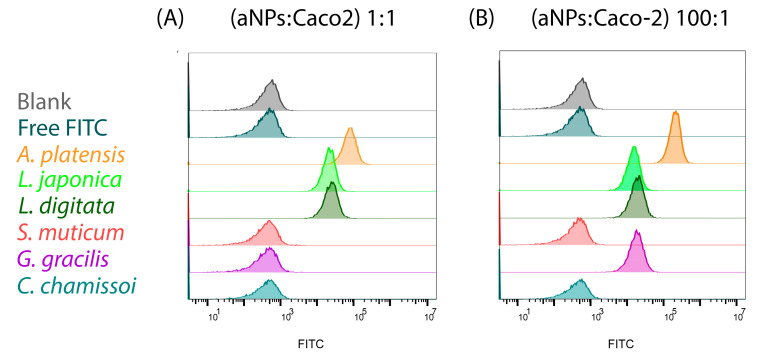
Cellular uptake into Caco-2 cells of aNPs encapsulating FD40 in two different incubation ratios of aNPs/Caco-2 of (**A**) 1:1 and (**B**) 100:1, respectively.

It is important to note that possessing high mucoadhesion force does not guarantee successful oral drug delivery. For example, oral DDSs must also be able to transverse the intestinal epithelium. Thus, we aimed to assess whether high mucoadhesion force correlates with enhanced cellular uptake. To this end, the aNPs mentioned above (highest and lowest mucoadhesion) were evaluated for their cellular uptake into Caco-2 cells (considered the standard in vitro model of the human intestinal epithelium) [23]. Caco-2 cells were exposed to the chosen aNPs encapsulating FD40 for three hours and then analyzed via a fluorescence-activated cell sorter (FACS) for their fluorescent content. The results are presented in Figure 7 and Table 4 below.

As shown in Figure 7 and Table 4, the aNPs with high mucoadhesion exhibited a 100% cellular uptake into Caco-2 cells when incubated at 1:1 and 100:1 ratios (aNPs: Caco-2 cells, Figure 7A). Conversely, the aNPs with low mucoadhesive forces—*S. muticum*, *G. gracilis*, and *C. chamissoi*—had no cellular uptake into Caco-2 cells when incubated at a 1:1 ratio (aNPs: Caco-2 cells). Interestingly, when incubated at a 100:1 ratio, *G. gracilis* NPs showed a 100% cellular uptake. At the same time, *S. muticum* and *C. chamissoi* were ineffective (Figure 7B). These results indicate a correlation between mucoadhesive force and cellular uptake in Caco-2 cells.

Further, the Mean Fluorescence Intensity of FITC (MFIF) in the exposed cells was also calculated and used for further analysis (Table 4). Herein, the measured MFIF of the more mucoadhesive aNPs was statistically more significant than that observed for the aNPs with low mucoadhesion. Moreover, *A. platensis* NPs exhibited an almost 3-fold increase in MFIF when incubated at a 100:1 ratio compared to 1:1 (73629 vs. 207996, respectively). Then again, this was not observed for *L. japonica* and *L. digitata* NPs, where there was decreased by 20–30% in MFIF when the incubation ratio was increased. We postulate that this might result from P-glycoprotein (Pgp) efflux, as it was found that the Pgp efflux transporter is expressed in Caco-2 cells [37]. However, *G. gracilis* NPs displayed a notable cellular uptake of 100% (and MFIF of 17103 at 100:1 incubation ratio), indicating that mucoadhesion is not the only parameter that affects cellular uptake, as shown before [38]. Moreover, based on the release experiments detailed in the Supporting Information, it is evidenced that the substance is retained within the NPs for nine days, underscoring the NPs’ capability to encapsulate the substance (Figure S3) sustainably.

## 3. Conclusions

In this study, we aimed to explore the potential of NPs derived from 14 different types of edible algae as innovative oral DDSs. First, we succeeded in producing NPs from the 14 different types of algae used in this study. All aNPs achieved the desired nanoscale size and exhibited a range of surface charges from negative to nearly neutral. In cryo-TEM photography, a spherical morphology was observed after lyophilization and re-sonication, which implies self-assembly, a characteristic of membrane lipids. Membrane lipids were detected via FTIR analysis. Among the tested aNPs, *A. platensis* NPs had the highest mucoadhesion in all three ex vivo animal models. No correlation was found between the groups, types, and protein content of aNPs and their mucoadhesion force. Moreover, the mucoadhesiveness of *A. platensis* NPs was not affected by exposure to pH levels typical in the small intestine. Generally, the highest mucoadhesion fracture strengths were observed in the intestines of mice. Additionally, the similarity between mouse and human Mucin2 sequences suggests that mouse intestines are a suitable model for predicting human mucoadhesion.

Next, the encapsulation efficiency of FD40 in A. platensis NPs reached 47%. In comparison, the release profile of FD40 showed no release of FD40 for nine days except for 7% release at the zero time point, indicating good protection of the encapsulant from the medium, which is essential for oral DDSs. Finally, the selected aNPs were tested for their cellular uptake into Caco-2 cells—the standard in vitro model of human epithelium—where a correlation was found between enhanced mucoadhesion and cellular uptake. In conclusion, we have shown that biomimetic aNPs have the potential to be an effective and innovative oral DDS, especially *A. platensis* NPs, which showed adequate size and surface charge, high yield, and high mucoadhesion and cellular uptake into Caco-2 cells. Moreover, the inherent sustainability, biocompatibility, biodegradability, and low immunogenicity of the biomimetic edible aNPs (e.g., *A. platensis*) increase their potential for use as oral DDSs. Yet, challenges remain, and further rigorous testing (e.g., in vivo experiments) is essential to assess these novel DDSs’ effectiveness fully.

## 4. Materials and Methods

### 4.1. Materials

Caco-2 cell line human was purchased from ATCC^®^ HTB-37™. Dulbecco’s Modified Eagle Medium (DMEM), L-Glutamine (L-Glu), and Penicillin–Streptomycin (P/S) were purchased from Sartorius, Israel. Phosphate-buffered saline (PBS), fetal bovine serum (FBS), Bradford reagent, trypsin, FD40, sucrose, and bovine serum albumin (BSA) were acquired from Sigma-Aldrich, Israel. All the edible algae were purchased from NutriCargo, USA (https://www.nutricargo.com accessed 1 February 2023) and received in powder form (Table 5).

### 4.2. Preparation of aNPs

To prepare aNPs, each algae powder (See Table 6) was weighed and dispersed in 80 mL double-distilled water. Subsequently, the algae solution was insonated (Q700, by Qsonica) in an ice bath using a Ø 1.3 cm transducer at 60% amplitude, 50% duty cycle (DC) for two minutes for cell lysis. After sonication, the solution was transferred to a centrifuge tube (Megafuge 16R, by Thermo Scientific, Kiryat Shemona, Israel) and centrifuged at 3200× *g* for 5 min at 4 °C. Then, the supernatant containing the lysate was subjected to another sonication cycle and centrifugation at the same conditions for downsizing. The supernatant containing aNPs was centrifuged at 10,000× *g* for 60 min at 4 °C. Later, the supernatant was gently poured over 2 mL of 60% sucrose followed by ultracentrifugation (Sorvall wX, by Thermo Scientific, Kiryat Shemona, Israel) at 200,000× *g* for 30 min at 4 °C. Then, 600 µL of solution was carefully removed from the layer above the 60% sucrose solution [8]. After production, the NPs were stored in a refrigerator at 4 °C.

### 4.3. Characterization of aNPs

The size, zeta potential, and concentration of aNPs were measured in water by a DLS instrument (Zetasizer Ultra Red, by Malvern Worcestershire, UK). The aNP yield was defined as the number of aNPs obtained divided by the initial algae mass. Additionally, protein content in aNPs was assayed via the Bradford method [39]. The sample was analyzed using an ELISA reader (Infinite M200 Tecan) at 595 nm. When the concentration fell outside the linear range, the sample was diluted to ensure accurate measurement within the linear range (i.e., 0–2 mg/mL). The concentration of BSA was calculated using the calibration curve (Figure S4).

TEM at cryogenic temperatures (Cryo-TEM) was used to image the produced aNPs. Vitrified specimens were prepared on a copper grid coated with a perforated lacey carbon 300 mesh (Ted Pella Inc. CA, USA). A 2.5 µL drop from the solution was applied to the grid and blotted with filter paper to form a thin liquid film. The blotted sample was immediately plunged into liquid ethane (−183 °C). This procedure was performed automatically in the Plunger (Leica EM GP). The vitrified specimens were then transferred into liquid nitrogen for storage. The samples were studied using the FEI Talos F200C TEM at 200 kV maintained at −180 °C, and images were recorded on an FEI Ceta 16M camera (4k × 4k CMOS sensor) at low-dose conditions to minimize electron beam radiation damage. The first example involved *A. platensis* NPs after production. In preparing the second sample, the same NPs were taken, and the sample underwent lyophilization, which involved freezing and subsequent water removal under vacuum conditions (FreeZone 2.5 L −84 °C Labconco Kansas City, MO, USA). The resulting lyophilized powder was resuspended in 1 mL of distilled water. Then, a mild sonication (30% amplitude, 50% duty cycle for 1 min) was applied in an ice bath.

The FTIR analysis method involved the preparation of a sample tablet containing 1 mg of *A. platensis* NPs after lyophilization and 99 mg of KBr. The components were thoroughly mixed using a mortar and pestle until a uniform powder was achieved. Subsequently, the powder was carefully compressed into tablets. The prepared tablet was placed in an FTIR instrument (FTIR-4600-type A, Jasco, Tokyo, Japan) using a standard light source, TGS detector, 32 accumulations, and at 4 cm^−1^ resolution.

### 4.4. Mucoadhesion Measurement of aNPs

Mucoadhesion measurements were conducted using a texture analyzer instrument (EZ-SX, by Shimadzu, Kyoto, Japan). Initially, 50 µL of the NPs solution was spread on a glass plate and dried for 30 min. Then, the small intestines of mice, pigs, or sheep were washed in PBS and carefully placed on top of a flat pin head (A = 40.7 mm^2^) so the lumen side faced outwards. The pin was mounted on the texture analyzer probe (capacity of 5 N, by Shimadzu), which was then lowered at a speed of 1.0 mm/s until the tissue came in contact with the aNPs with an applied force of 20 mN and 200 mN for 420 s [40]. The contact forces were chosen according to a study conducted to measure the peristaltic forces inside the intestines of a lamb. These forces were measured via an encapsulated prototype with a force sensor, where it was found that the range of peristaltic forces in the small intestine was between 0–180 mN [41]. As mentioned, the mucoadhesive fracture strength was derived from the peak force required to separate two layers [40]. In addition, the mucoadhesion forces of *A. platensis* NPs were measured at three pH values characteristic of different parts of the small intestine, and the zeta potential was tested for each pH. Everything was tested against pig intestinal tissue and is shown in Table S1.

### 4.5. Encapsulation of Fluorescent Molecule in aNPs

For these experiments, six algae were selected—three with the most significant mucoadhesive forces and three exhibiting the lowest mucoadhesive forces. To evaluate the cellular uptake in Caco-2 cells, FD40 was encapsulated in the chosen aNPs. First, 2 mL of the aNPs was mixed with 0.1 mL of 0.5 mg/mL FD40 and 7.4 mL of PBS. Then, the mixture was placed in an ice bath and sonicated using a microtip (Ø 0.3 cm) ultrasound transducer at 60% amplitude and 75% DC for two minutes. After sonication, the solution was refrigerated for 10 min. The sonication and refrigeration were repeated one more time. For the measurement of encapsulation efficiency, first, 200 µL of the (final) supernatant was transferred into a 96-well flat black plate (by Greiner, Monroe, NC, USA) to determine the amount of free FD40 and analyzed using a fluorometer (Infinite M200 Tecan) at excitation and emission wavelengths of 490 nm and 525 nm, respectively. The concentration of FD40 in the supernatant was calculated using the calibration curve (Figure S5).

### 4.6. Release profile of FD40 from A. platensis NPs

To assess the release profile from aNP, 12 mL of a solution containing aNPs from *A. platensis* encapsulating FD40 in PBS was placed in a 20 mL scintillation vial. The vial was covered with aluminum foil to minimize photobleaching. For nine consecutive days, at 24 h intervals, the mixture was ultracentrifuged at 200,000× *g* for 10 min at 4 °C. After each centrifugation, 200 µL of the supernatant was collected and replenished with an equivalent volume of PBS. The concentration of FD40 in the supernatant was calculated using the calibration curve (Figure S5).

### 4.7. Protein BLAST Analysis

The Mucin2 glycoprotein serves as the primary constituent of the outer mucus layer and imparts viscoelastic properties to mucus [42]. The human Mucin2 glycoprotein sequence was retrieved from the Entrez repository with the accession number AZL49145.1 [43] in FASTA format. The mouse, pig, and sheep sequences were obtained from the Protein Basic Local Alignment Search Tool (BLAST) (accessions are shown in Table 2). Later, the Protein BLAST [44] was used to search for similarities against the sequences of Mucin2 from mice, pigs, and sheep. The presented score value was used to assess the degree of similarity between the different mucin sequences.

### 4.8. Caco-2 Uptake of aNPs

To prepare the cell culture medium, 450 mL of DMEM was thoroughly mixed with 50 mL of FBS, 5 mL of L-Glu, and 5 mL of P/S. The cellular uptake of the chosen aNPs was evaluated with Caco-2 cells—the prominent GI in vitro model. First, Caco-2 cells were seeded in 24-well plates (Greiner) and incubated at a concentration of 2 × 10^5^ cells/mL for 48 h at 37 °C in 1 mL of cell medium. Then, the cells were counted using an automated cell counter (A_2_S, b EVE™, Waltham, MA, USA), and the number of NPs was determined using the DLS instrument. Following these measurements, the ratios 1:1 and 100:1 (aNPs/Caco-2 cells) were used for each tested alga. Caco-2 cells were incubated with free FD40 at an equivalent amount as encapsulated in the aNPs and used as a control. After three hours of incubation, the wells were carefully washed twice with PBS to remove free FD40 and aNPs. Subsequently, 300 µL of trypsin was added to each well and incubated for five minutes in the incubator to detach the cells. Then, the cell suspension was transferred to an Eppendorf test tube with 1 mL of medium. Cells were obtained by centrifugation at 500× *g* for 12 min and were resuspended in a solution of PBS containing 0.5% BSA. Later, the samples were transferred to 96-well microplates and analyzed under the FACS instrument (CytoFLEX Beckman Coulter, Indianapolis, IN, USA) to test the degree of internalization of Caco-2 cells to algae NPs.

### 4.9. Statistical Analysis

The data were analyzed using Prism version 9 (by GraphPad, Boston, Massachusetts, USA). The normality of the data was assessed using the Shapiro–Wilk test. The Brown–Forsythe test was used to confirm the homogeneity of variances. A one-way ANOVA (two-tailed) was employed to examine differences among multiple groups using the Bonferroni post hoc test. The data in the study represents the mean and standard deviation (SD) of n ≥ 3. All statistical analyses were conducted at a significance level of α = 0.05.

## Figures and Tables

**Table 1:** The obtained size, polydispersity index (PDI), and protein content of the NPs produced from algae.

Type of aNPs	Size (nm)	PDI	Relative NP Concentration *	Protein Content (mg/mL)
*C. chamissoi*	605 ± 67	0.22 ± 0.02	1.5 ± 0.3	0.04 ± 0.01
*G. gracilis*	232 ± 13	0.24 ± 0.02	9.1 ± 1.2	0.07 ± 0.001
*C. crispus*	275 ± 24	0.33 ± 0.03	4.1 ± 1.0	0.01 ± 0.001
*U. pinnatifida*	466 ± 18	0.29 ± 0.01	6.4 ± 0.5	0.44 ± 0.01
*S. muticum*	170 ± 07	0.24 ± 0.01	28.2 ± 9.9	0.68 ± 0.06
*L. digitata*	212 ± 20	0.27 ± 0.01	12.4 ± 7.8	0.11 ± 0.001
*L. japonica*	217 ± 10	0.19 ± 0.02	40.0 ± 8.7	0.68 ± 0.001
*E. cava*	235 ± 12	0.19 ± 0.01	27.3 ± 8.0	0.81 ± 0.01
*H. pluvialis*	251 ± 07	0.21 ± 0.01	30.2 ± 3.9	3.24 ± 0.02
*M. pyrifera*	252 ± 20	0.29 ± 0.03	6.7 ± 1.0	0.15 ± 0.02
*P. palmata*	245 ± 33	0.44 ± 0.12	7.1 ± 0.4	0.77 ± 0.09
*G. skottsbergii*	381 ± 13	0.48 ± 0.01	3.6 ± 0.2	0.01 ± 0.01
*A. platensis*	126 ± 02	0.14 ± 0.001	60.8 ± 2.9	2.61 ± 0.81
*C. vulgaris*	157 ± 11	0.25 ± 0.05	5.1 ± 0.5	0.80 ± 0.04

Note: Values represent the average ± SD of at least three repetitions. * × 10^9^ NPs/(mL × g).

**Table 2:** Comparative analysis using the Protein BLAST tool of the sequences of Mucin2 glycoprotein from a mouse, sheep, and a pig compared to the sequence of human Mucin2.

Animal	Sequence ID	Length [aa]	Identities [%]	Positives [%]	Gaps [%]	Query Cover [%]	Score
Mouse	NP_076055.4	4576	80	87	0	43	2532
Sheep	XP_042093899.1	5972	49	63	3	87	1495
Pig	XP_020938146.1	5759	49	63	3	60	1474

**Table 3:** *EE* of tested aNPs loaded with FD40.

Type of aNPs	*EE*
*A. platensis*	47%
*L. japonica*	55%
*L. digitata*	40%
*S. muticum*	32%
*G. gracilis*	35%
*C. chamissoi*	34%

**Table 4:** Mean Fluorescence Intensity of FITC (MFIF) and percentage of cellular uptake into Caco-2 cells of the tested aNPs encapsulating FD40 for 1:1 and 100:1 incubation ratios of aNPs to Caco-2 cells, respectively.

Type of aNP	MFIF 1:1	MFIF 100:1	Uptake 1:1	Uptake 100:1
Free FITC	228	228	0.59%	0.59%
*A. platensis*	73,629	207996	100%	100%
*L. japonica*	20,984	14238	100%	100%
*L. digitata*	24,576	17838	100%	100%
*S. muticum*	229	212	0.59%	0.54%
*G. gracilis*	229	17103	0.59%	100%
*C. chamissoi*	255	270	0.65%	0.69%

**Table 5:** General information about the 14 tested algae.

Common Name	Botanical Name	Catalog #	Group-Type
*Chondracanthus chamissoi*	*Chondracanthus chamissoi*	ncchchpwd	Red macroalgae
*Gracilaria*	*Gracilaria gracilis*	FRX730	Red macroalgae
*Irish moss*	*Chondrus crispus*	FRX853	Red macroalgae
*Wakame*	*Undaria pinnatifida*	FRX1419	Brown macroalgae
*Sargassum seaweed*	*Sargassum muticum*	ncsasepwd	Brown macroalgae
*Kelp laminaria digitata*	*Laminara digitata*	FRX874PAC	Brown macroalgae
*Kombu*	*Laminaria japonica*	FRX886	Brown macroalgae
*Ecklonia cava*	*Ecklonia cava*	FRX3544	Brown macroalgae
*Astaxanthin*	*Haematococcus pluvialis*	FRX177	Red microalgae
*Giant kelp*	*Macrocytis pyrifera*	ncgikepwd	Brown macroalgae
*Dulse*	*Palmaria palmata*	FRX576	Red macroalgae
*Gigartina red marine*	*Gigartina skottsbergii*	FRX680	Red macroalgae
*Spirulina*	*Arthospira platensis*	FRX1333	Green microalgae
*Chlorella vulgaris*	*Chlorella vulgaris*	FRX454	Green microalgae

**Table 6:** Initial algal powder mass used for the production of aNPs.

Type of Algae	Initial Mass * (g)
*C. chamissoi*	2.0
*G. gracilis*	2.0
*C. crispus*	0.5
*U. pinnatifida*	2.0
*S. muticum*	2.0
*L. digitata*	2.0
*L. japonica*	2.0
*E. cava*	2.0
*H. pluvialis*	2.0
*M. pyrifera*	2.0
*P. palmata*	2.0
*G. skottsbergii*	0.3
*A. platensis*	0.2
*C. vulgaris*	0.4

* The initial amount of each algal powder was determined after several trial-and-error experiments.

## Data Availability

All the data acquired and used in this manuscript is available on request to the corresponding author. The data are contained within the article and/or are available from the corresponding author upon reasonable request.

## Supplementary Materials

The following supporting information can be downloaded at https://www.mdpi.com/article/10.3390/md22030098/s1, Figure S1:The hydrodynamic diameter size distribution (intensity wise) of the tested aNPs.; Figure S2: FTIR interferogram (32 scans, 2 cm^−1^ resolutions) of *A. platensis* NPs sample. Blue arrows point to the distinct peaks of membrane lipids. Figure S3: The actual accumulated percentage release profile of FD40 from *A. platensis* NPs; Figure S4: Bradford calibration curve, correlating BSA protein concentration with absorbance at 595 nm. Data is averaged from three triplicates, with statistical tests conducted at a significance level of α = 0.05 for normal distribution and linear regression assessment. The values represent the mean ± SD of n = 3; Figure S5: FD40 calibration curve, correlating FD40 concentration in PBS with relative fluorescent units (RFU) at 525 nm. Data is averaged from three triplicates, with statistical tests conducted at a significance level of α = 0.05 for normal distribution and linear regression assessment. The values represent the mean ± SD of n = 3; Table S1: Mucoadhesion forces (against porcine intestinal tissue) and zeta potential of *A. platensis* NPs at various small intestinal pH levels of humans after fasting [45].
