# Supplementary material for: Algae-Based Nanoparticles for Oral Drug Delivery Systems
Source: Mar Drugs. 2024 Feb 21;22(3):98. doi: 10.3390/md22030098 (PMC10971847; doi:10.3390/md22030098)
Supplement: Supplementary file 1 [file marinedrugs-22-00098-s001.zip › marinedrugs-2874760-supplementary.pdf]

Supporting Information

Algal-Based Nanocarriers as Oral Drug Delivery Systems

Eliyahu Drori, Dhaval Patel, Sarah Coopersmith, Valeria Rahamim, Chen Drori, Suchita Suryakant Jadhav, Roni Avital, Yaakov Anker and Aharon Azagury *

Department of Chemical Engineering, Ariel University, Kiryat Hamada 3, Ariel 4070000, Israel; elidrori@gmail.com (E.D.); dhaval30103@gmail.com (D.P.); sarahchouraqui63@gmail.com (S.C.); valeria.rahamim@gmail.com (V.R.); chendrori27@gmail.com (C.D.); jadhavs@ariel.ac.il (S.S.J.); roni.avital484@gmail.com (R.A.); kobia@ariel.ac.il (Y.A.)
* Correspondence: aharona@ariel.ac.il

The size distribution of the aNPs for each alga obtained from the DLS instrument is presented in Figure S1 below.

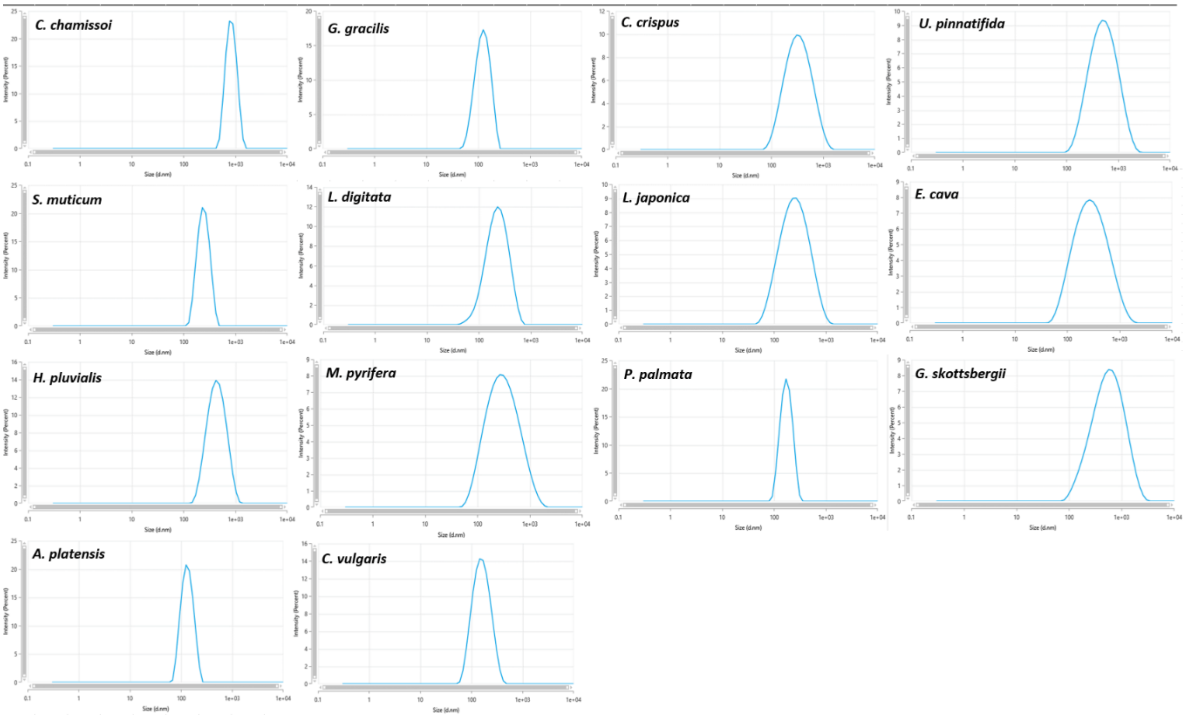

Figure S1. The hydrodynamic diameter size distribution (intensity wise) of the tested aNPs.

A singular peak was detected in each of the analyzed aNPs, indicating the presence of a single population.

FTIR analysis was used to detect lipid content in aNPs, as shown in Figure S2 below.

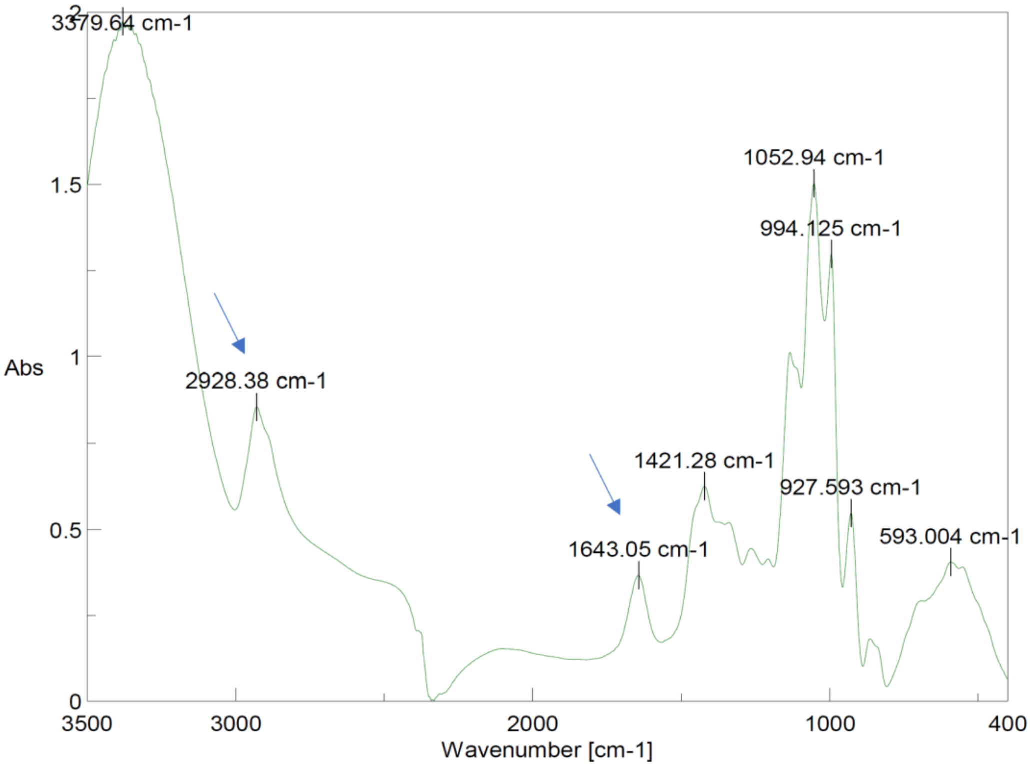

Figure S2. FTIR interferogram (32 scans, 2 cm^{-1} resolutions) of *A. platensis* NPs sample. Blue arrows point to the distinct peaks of membrane lipids.

The detection of membrane lipids via FTIR analysis was based on previous research by Portaccio et al.[1]. They found that membrane lipids exhibit two distinct peaks at 2800-3100 cm^{-1} and slightly below 1800 cm^{-1}. As shown in Figure S2, the FTIR interferogram of *A. platensis* aNPs also exhibits these two peaks, indicating the presence of membrane lipids. This observation further supports our claim that aNPs possess a liposomal structure.

Table S1. Mucoadhesion forces (against porcine intestinal tissue) and zeta potential of A. platensis NPs at various small intestinal pH levels of humans after fasting**.**

Small intestine section	pH	Fracture strength [μN/mm²]	Zeta potentials [mV]
Duodenum	6.1	1894 ± 101	-39 ± 1.8
Jejunum	5.4	1954 ± 125	-37 ± 1.6
Ileum	7.8	1967 ± 117	-39 ± 2.5

These results showed no statistically significant change in the measured mucoadhesion or ZP of aNPs in different pH ranges of 5.4 to 7.8. Thus, we can conclude that the intestinal pH range does not affect the mucoadhesion of aNPs. As for the type of interaction of the aNPs with mucosal layers, the stable ZP could indicate either non-covalent interactions: electrostatic, VDW, hydrogen bonds, or hydrophobic interactions. Another support for electrostatic connections between the aNPs and mucin is that the negative aNPs can stick to the charged amino acids in the terminal domains [2].

Next, the actual accumulated percentage release of FD40 from *A. platensis* NPs was tracked over nine days, as depicted in Figure S3 below. Note that the percentage released was calculated based on the actual encapsulation efficiency of FD40 in *A. platensis* NPs.

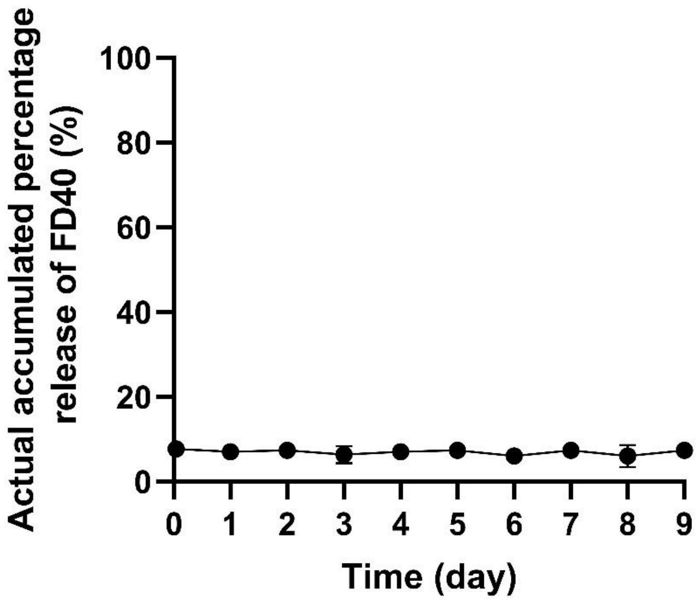

Figure S3. The actual accumulated percentage release profile of FD40 from *A. platensis* NPs

Figure S3 illustrates the actual accumulated percentage release profile of FD40 from *A. platensis* NPs over nine days. As seen, there is a minor release of approximately 7% at the zero-time point, after which the accumulated release plateaued, indicating no additional release of FD40. This result suggests that *A. platensis* NPs can potentially protect the encapsulant from the harsh enzymatic degradation environment in the GI tract.

The calibration curves used in this study are presented below in Figures S4 and S5.

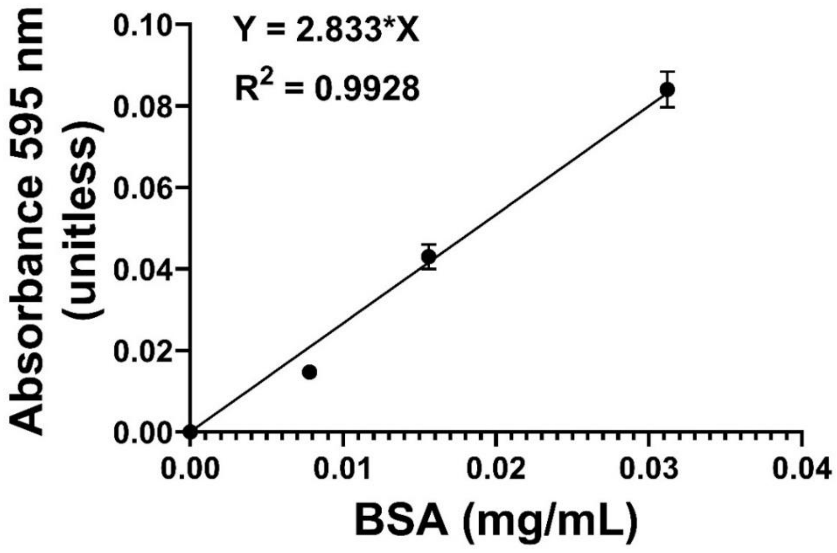

Figure S4. Bradford calibration curve, correlating BSA protein concentration with absorbance at 595 nm. Data is averaged from three triplicates, with statistical tests conducted at a significance level

of $\alpha = 0.05$ for normal distribution and linear regression assessment. The values represent the mean ± SD of n=3.

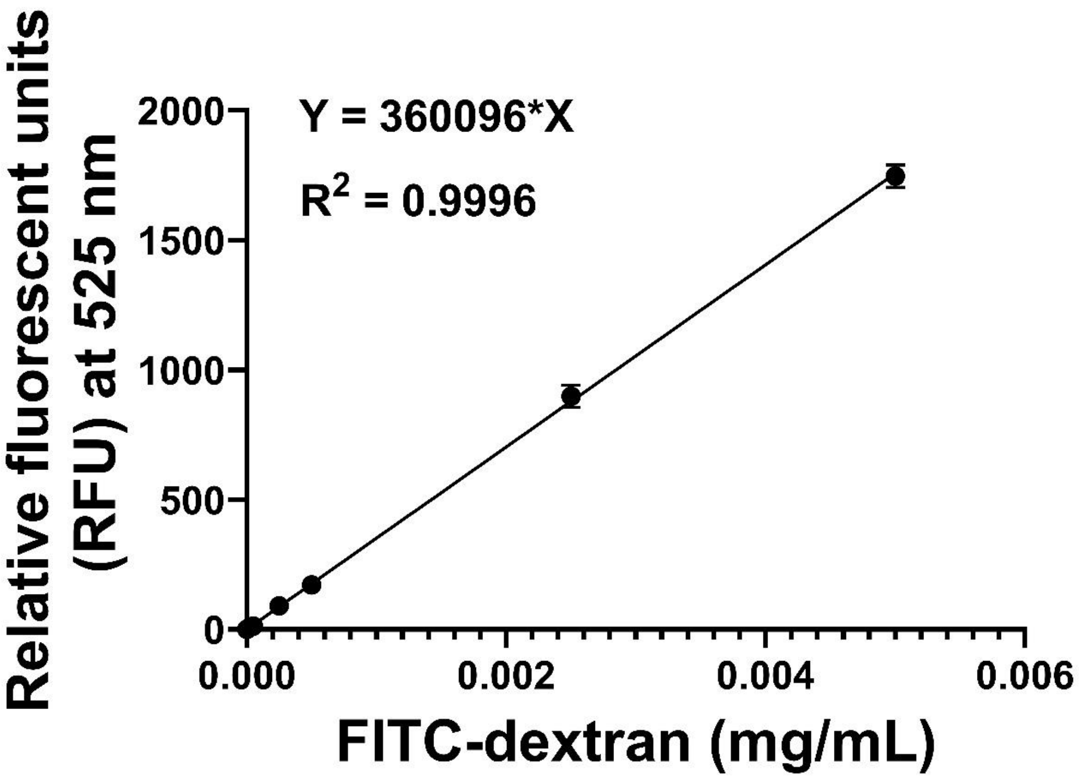

Figure S5. FD40 calibration curve, correlating FD40 concentration in PBS with relative fluorescent units (RFU) at 525 nm. Data is averaged from three triplicates, with statistical tests conducted at a significance level of $\alpha = 0.05$ for normal distribution and linear regression assessment. The values represent the mean ± SD of n=3.

References

1. Portaccio, M.; Faramarzi, B.; Lepore, M. Probing Biochemical Differences in Lipid Components of Human Cells by Means of ATR-FTIR Spectroscopy. *Biophysica* **2023**, *3*, 524–538, doi:10.3390/biophysica3030035.
2. Subramanian, P. Mucoadhesive Delivery System: A Smart Way to Improve Bioavailability of Nutraceuticals. *Foods* **2021**, *10*, doi:10.3390/foods10061362.
